# Spesolimab in generalized pustular psoriasis complicated by acrodermatitis continua of Hallopeau: a case report and mechanistic insights

**DOI:** 10.3389/fimmu.2025.1563553

**Published:** 2025-05-15

**Authors:** Xin-Yi Hou, Hai-Lu Xiao, Jing-Yu Wang, Jin Zhang, Bo Ren, Chu-chu Niu, Fei-Fei Liu, Bin Lu

**Affiliations:** ^1^ Department of Dermatology, Affiliated Hospital of Jining Medical University, Jining, Shandong, China; ^2^ School of Clinical Medicine, Jining Medical University, Jining, Shandong, China; ^3^ Department of Pathology, Affiliated Hospital of Jining Medical University, Jining, Shandong, China

**Keywords:** spesolimab, generalized pustular psoriasis, acrodermatitis continua of Hallopeau, IL-36 pathway, IL-1

## Abstract

A 62-year-old Chinese woman presented to the hospital for help with generalized pustular psoriasis (GPP) and acrodermatitis continua of Hallopeau (ACH). While conventional treatments failed to achieve significant improvement, the patient received two doses of spesolimab, and the effect was remarkable. No adverse reactions were observed in the follow-up period.

## Highlights

- A patient who suffered from generalized pustular psoriasis (GPP) coexisting with acrodermatitis continua of Hallopeau (ACH) exhibited no significant clinical improvement following conventional therapies, however, an effect was observed in the patient after the injection of spesolimab for the first time and signs and symptoms were quickly brought under control.
- Spesolimab, by directly blocking the receptor with a therapeutic antibody, is the first biologic agent that works via the IL-36 pathway, which is associated with the pathogenesis of GPP. Thus, spesolimab delivers more precise treatment.
- The patient remained free of adverse reactions during the post-treatment observation period until 10 March 2025.
- There are few reports about the use of spesolimab in patients with ACH coexisting with GPP. Our report provides a case reference for such patients, which has practical significance.

## Introduction

Generalized pustular psoriasis (GPP) is an uncommon and intractable variant of the disorder characterized by diffuse erythematous rashes and recurrent pustular flares (1). It is hard to treat this disease because of a paucity of reliably efficacious therapeutics. It has been indicated that the interleukin (IL)-36 pathway plays an important role in the occurrence and development of this disease (2). Thus, IL-36 receptor antagonists are emerging as a promising therapy for pustular psoriasis, providing a rapid and efficacious response. Herein we share our experience with spesolimab in a Chinese patient with GPP and acrodermatitis continua of Hallopeau (ACH) who was heavily treated with other common methods with poor response.

## Case report

A 62-year-old female patient presented to our department in August 2024 with the symptom of a generalized pustular eruption that appeared 20 days previously. The patient had a past medical history of ACH for 16 years. The rash of the patient progressed rapidly, with severe skin tenderness. She was admitted to the inpatient department. At the time of hospital admission, the symptoms of ACH remained persistent.

**Figure 1:**
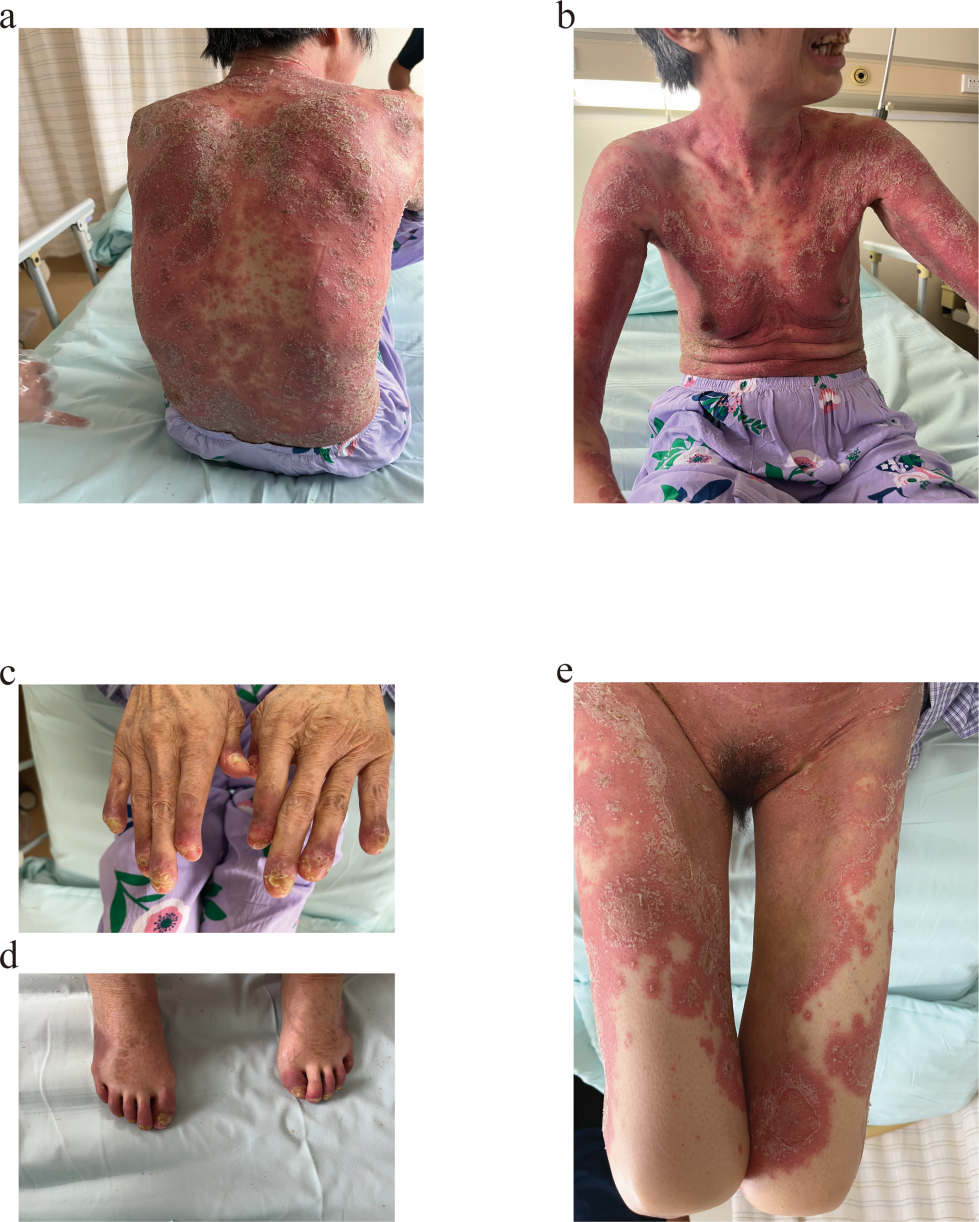
Skin lesions of the patient at first admission, the lesions manifest as erythematous plaques covered with pustules, with extensive confluence leading to widespread erythema, scaling, and pustular lakes. Nail plates are dystrophic, showing subungual pustules. **(a, b)** Trunk. **(c, d)** Distal extremities. **(e)** Perineum and bilateral lower limbs.

The physical examination was notable for widespread, red plaques studded with pustules, many coalescing into broad areas of erythema, scaling, and pustules on the trunk and extremities. Swelling and deformity could be observed in the finger joints and toe joints. The nails and toenails were deformed, and there were pustules under the nail plate and tenderness on the skin, which was especially obvious when walking (**Figure 1**). Standardized scoring systems were utilized to assess disease severity. The score for Pustular Psoriasis Area and Severity Index (GPPASI) was 64.8, Nail psoriasis Area and Severity Index (NPASI) was 8, Dermatology Quality of Life Index (DLQI) was 30, BSA was 80%, and Pustular Psoriasis Physician’s Global Assessment (GPPGA) was 4.

**Figure 2:**
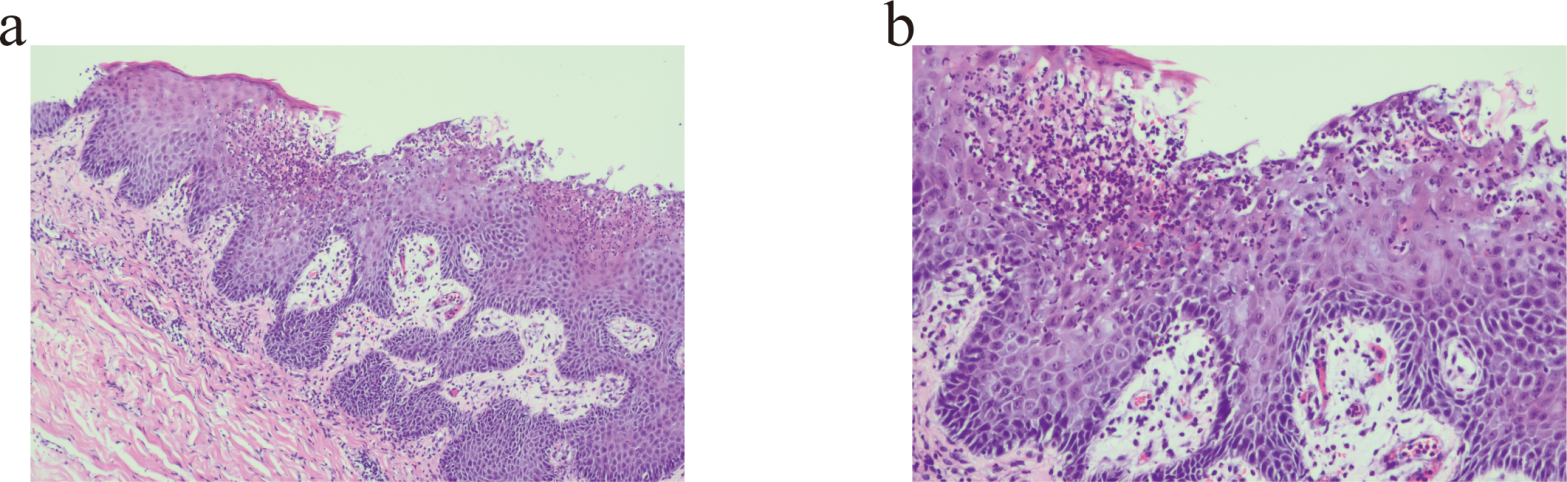
Pustule formation was observed in the epidermis, and numerous neutrophil infiltrations were observed. **(a)** Haematoxylin and eosin, original magnification ×100. **(b)** Haematoxylin and eosin, original magnification ×200.

A complete study was performed with blood and skin cultures. No evidence of infectious diseases, such as syphilis and human immunodeficiency virus (HIV), was found by blood testing. Leukocytosis and neutrophilia with elevated phase reactants were revealed. There was no specific abnormality in the results of her echocardiogram and a body CT scan. Skin biopsies were reported as diagnostic for pustular psoriasis (**Figure 2**). Microbiological samples were negative.

**Figure 3:**
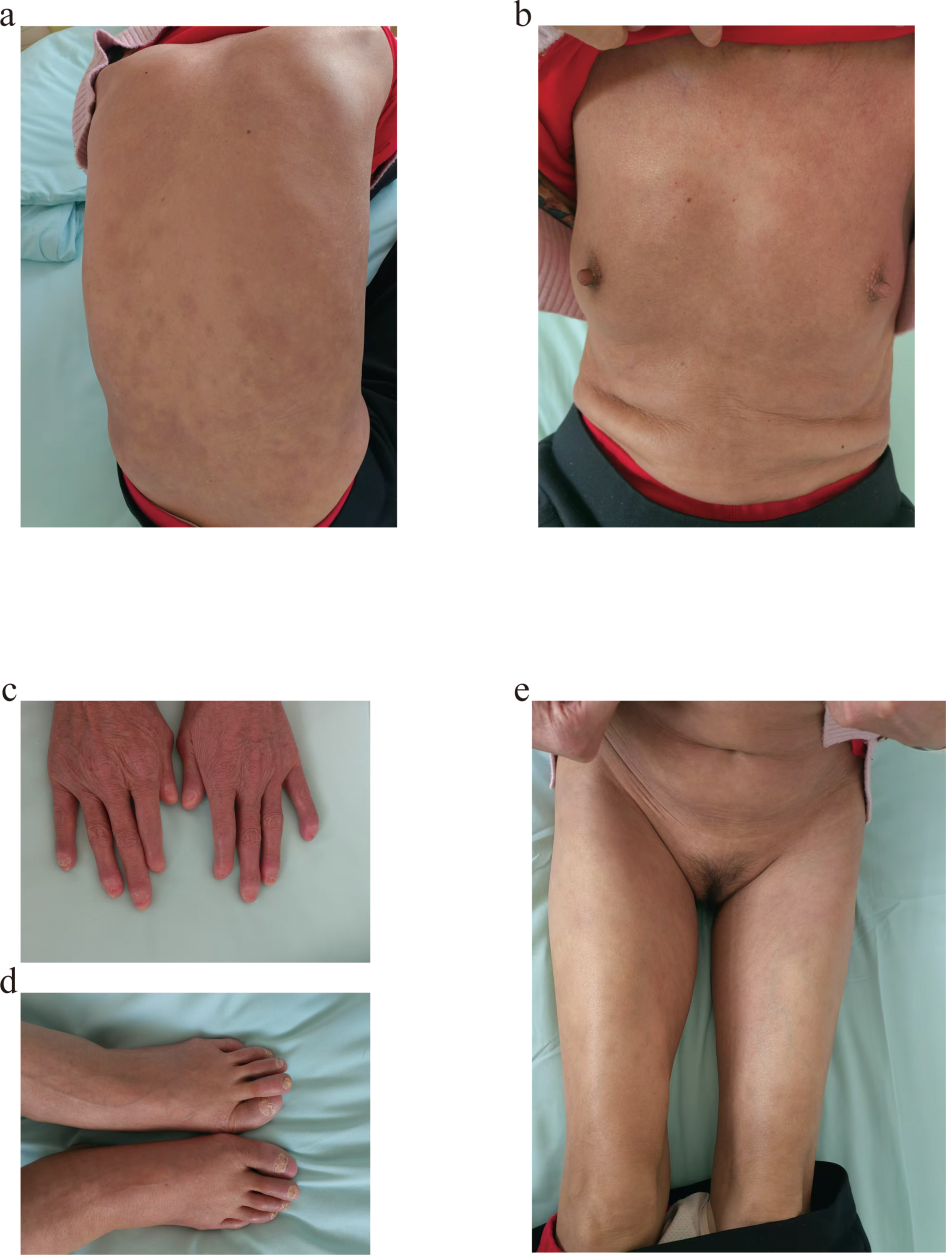
Before using Spesolimab for the second time,notable resolution of the skin lesions was documented. **(a, b)** Trunk. **(c, d)** Distal extremities. **(e)** Perineum and bilateral lower limbs.

The diagnosis of GPP was definitively established based on the patient’s medical history, clinical presentation, and histopathological findings. The patient was administered polyene phosphatidylcholine, aminopeptide, and acitretin for 1 month, however, the medication treatment failed to achieve a significant improvement. In order to further improve the patient’s health, she was treated with a single intravenous infusion of 900mg of spesolimab. A remarkable response to spesolimab with an almost complete resolution of pustules in 6 hours in the patient was observed. There were no adverse reactions in the follow-up period. After 2 weeks, at the patient’s follow-up in an outpatient dermatology clinic, there were no visible lesions but patchy pigmentation. Another 2 weeks later, the patient was readmitted to the hospital for treatment with spesolimab in order to consolidate the effects and prevent GPP recurrence (**Figure 3**). During the 6-month follow-up period, no recurrence was observed, and no adverse events were reported. The long-term efficacy still needs further observation.

## Discussion

As an autoinflammatory skin disease, the notable features of GPP are eruption of sterile pustules with or without severe systemic symptoms, such as organ failure and sepsis. ACH, a rare dermatological disorder, presents as a sterile pustular eruption localized to one or more digits and is characterized by a chronic clinical progression. In terms of classification, ACH is classified as a localized pustular psoriasis (3). ACH demonstrates a characteristically chronic and treatment-resistant clinical course, with spontaneous remission seldom documented in medical literature (4). It is difficult to achieve significant results in both of these diseases with treatment.

GPP coexisting with ACH is hard to treat because the condition is so rare, resulting in an absence of standardized treatment guidelines. Current therapeutic strategies for ACH largely mirror those employed for other psoriasis subtypes, reflecting shared pathogenic mechanisms within the psoriasis spectrum (5). Methotrexate, cyclosporine, and acitretin are systemic drugs commonly used in GPP and ACH. However, the effects of these treatments are often not significant and can be associated with a multitude of adverse effects, including hypertension, renal toxicity, and teratogenicity (6), and patients often relapse.

A lack of extremely effective therapies poses serious challenges for GPP and ACH management for physicians. The fundamental reason is that the etiology of GPP and ACH remains to be fully elucidated. The development of GPP and ACH is complex, and there is a significant overlap in the pathogenic mechanisms between GPP and ACH. The IL-36 pathway has been identified to play a key role in the pathogenesis of the disorder (7). GPP and ACH may occur due to overexpression of IL‐36 and the loss-of-function mutation in the IL-36 receptor antagonist (IL-36RA), which leads to increased levels of pro-inflammatory cytokines and activation of IL-36 receptors in skin cells (8–10). IL-36RN, a negative regulator of the IL-36 pathway, encodes the IL-36RA, and in some sporadic and familial cases of GPP, an IL36RN gene mutation has been detected (1, 11, 12), mainly in those not associated with plaque psoriasis (PP). Thus, anti-IL-36R drugs, such as spesolimab, can be effective in treating GPP coexisting with ACH. Spesolimab is a humanized monoclonal antibody that inhibits the ability of IL-36 to bind and initiate proinflammatory cascades by blocking the IL-36 receptor (6). Treatment guidelines have indicated that the recommended grade for IL-36 receptor antagonists, such as spesolimab, is C. Spesolimab has had the broadest clinical development with five phase-I and three phase-II trials. In phase I, spesolimab completed clearance at week 4, and this was maintained at week 12 regardless of the presence of IL36RA mutation (2), which proved good efficacy. In phase II, 54% of patients were free of pustules at week 1, and 60% had complete clearance after 1–2 doses, with some patients having a complete clearance in less than 24 h. Furthermore, a sustained response and a favorable safety profile were found at week 12 of treatment (13). In previous reports, most of the patients using spesolimab achieved the desired treatment results, however, a small number of patients showed adverse drug reactions. Common adverse reactions are rash, nasopharyngitis, headache, and acne (14).

Although our study highlights the central role of IL-36 signaling in this patient, the interplay between the IL-1 and IL-36 pathways in GPP pathogenesis merits attention. The release of excessive amounts of IL-1 and IL-36 results in an inflammatory response in the skin, which leads to the development of pustules in pustular psoriasis. Targeted therapies, such as IL-1 antagonists, have demonstrated definitive efficacy in improving the prognosis of pustular eruptions following COVID-19 vaccination (15). However, further research is required to elucidate the mechanistic interplay between IL-1 and IL-36 in pustule formation, thereby providing critical insights to guide future investigative directions.

Although there have been many cases of spesolimab treating GPP, there are few reports on the use of spesolimab in GPP coexisting with ACH. The patient in this case report suffered from GPP with ACH for a long period of time. She received many treatments; however, clinical improvement was not remarkable. An effective manifestation was observed in the patient after the injection of spesolimab for the first time. Her signs and symptoms were quickly brought under control, the rash disappeared except for the lesions on the fingers and toes, and no adverse effects were observed. We evaluated the disease severity again after the first treatment with spesolimab, and the score of GPPASI was 0, NPASI was 4, DLQI was 5, BSA was 0, and GPPGA was 0. To improve the treatment effect, she received the treatment with spesolimab again after 1 month; however, the long-term benefit of the therapy needs further observation, including safety and tolerability profile.

## Conclusion

When a generalized pustular eruption emerges, the diagnosis of GPP should be considered. This case highlights the efficacy of IL-36 inhibitors, such as spesolimab, for GPP coexisting with ACH. The approach of targeting proinflammatory cytokines not only shows a rapid and effective response but also minimizes the adverse effects. This is a potential treatment modality that should be considered when other treatments are not effective.

## Data Availability

The original contributions presented in the study are included in the article/supplementary material, further inquiries can be directed to the corresponding author/s.
